# Successful application of large microneedle patches by human volunteers

**DOI:** 10.1016/j.ijpharm.2017.02.011

**Published:** 2017-04-15

**Authors:** Anastasia Ripolin, James Quinn, Eneko Larrañeta, Eva Maria Vicente-Perez, Johanne Barry, Ryan F. Donnelly

**Affiliations:** School of Pharmacy, Queen’s University Belfast, 97 Lisburn Road, Belfast BT9 7BL, UK

**Keywords:** Microneedles, Self-application, Large patches, Clinical translation

## Abstract

We describe, for the first time, the design, production and evaluation of large microneedle patches. Such systems, based on 16 individual microneedle arrays (needle height 600 μm), were prepared from aqueous blends of 15% w/w Gantrez^®^ S97 and 7.5% w/w poly(ethyleneglycol) 10,000 Da. Ester-based crosslinking was confirmed by FTIR and mechanical strength was good. Insertion depths in a validated skin model were approximately 500 μm. Ten human volunteers successfully self-inserted the microneedles of these larger patches in their skin, following appropriate instruction, as confirmed by transepidermal water loss measurements. The mean insertion depth ranged between 300 and 450 μm over the area of the large patches. That this was not significantly different to a single unit MN patch self-applied by the same volunteers is encouraging. Microneedle patch sizes much larger than the 1–2 cm^2^ will be required if this technology is to be successfully translated to clinic for delivery of drug substances. The work described here suggests that use of such larger patches by patients can be successful, potentially opening up the possibility for a significant expansion of the size of the market for transdermal drug delivery.

## Graphical abstract

**Figure:**
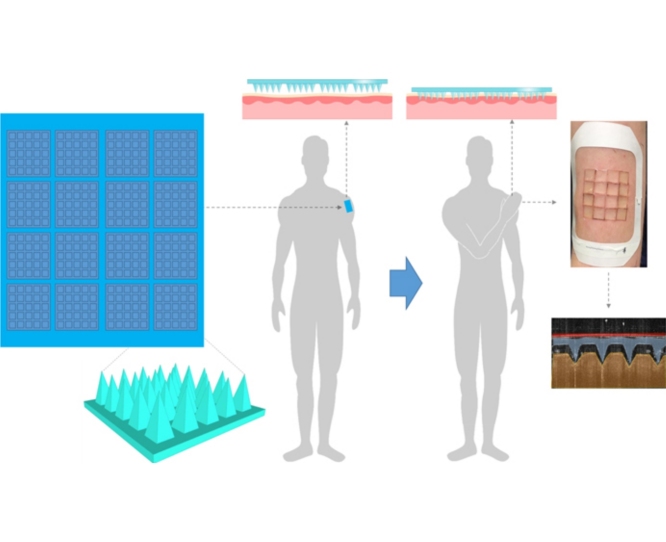

## 1 Introduction

Microneedle (MN) arrays are minimally-invasive devices that are used to penetrate the skin’s outermost layer, the *stratum corneum* (SC), which is the principal barrier to topically-applied drugs (Donnelly et al., 2012b, Larrañeta et al., 2016c, Prausnitz, 2004). MN arrays are currently of great interest to the pharmaceutical industry, due to the number of advantages they possess over traditional methods of drug delivery, such as oral and parenteral administration. Some of the benefits include the ability to painlessly administer the active pharmaceutical ingredient, bypass of hepatic first pass metabolism and extension of the range of types of drug that can be delivered intradermally and transdermally (Tuan-Mahmood et al., 2013).

A wide variety of substances have been successfully delivered transdermally and intradermally using MN arrays (Donnelly et al., 2012a, Larrañeta et al., 2016a, Prausnitz, 2004, Tuan-Mahmood et al., 2013). However, the predominant focus in the field has been on vaccines (Henry et al., 1998, McGrath et al., 2011). This is hardly surprising, given the potential for stable, dry state formulation, the avoidance of needle-stick injuries common with hypodermic syringes, dose-sparing through direct targeting of viable skin’s abundance of professional antigen-presenting cells and the self-disabling nature of dissolving MN. Indeed, MN vaccines have the potential to revolutionise vaccination, especially in the developing world. In studies where delivery of therapeutic drug substances using has been exemplified, the focus has tended to be on illustration of the capability of MN in delivering a substance with particular physicochemical characteristics and little mention is typically made of the actual amount delivered or its relevance to therapeutic human doses.

Vaccines tend to be quite potent and so delivery of even microgram quantities of antigen, antigen/adjuvant combination, virus-like particle or even DNA is often sufficient to elicit an immune response, especially when targeted to the viable epidermis and/or dermis. This means that small, postage stamp-sized MN patches that can be inserted into skin by fairly gentle thumb pressure are sufficient to achieve successful vaccination. Our own work has focused strongly on delivery of therapeutically-relevant doses of drugs using MN patches (Donnelly et al., 2012a, Donnelly et al., 2014b, Kearney et al., 2016). We have measured plasma levels in animal models and extrapolated to estimate suitable patch sizes for achievement of therapeutic plasma levels in humans. Since most commonly-used small molecule drugs tend to require oral doses in the range of tens to hundreds of milligrams daily, the patch sizes we have estimated have ranged from 10 cm^2^–30 cm^2^. Such patch sizes are well within the range of marketed transdermal patches. Indeed, Novartis market Nicotinell^®^ (nicotine) patches of 30 cm^2^ (Novartis, 2017), while Janssen market Duragesic^®^ CII (fentanyl) patches of 32 and 42 cm^2^ (Janssen, 2017), for example. MN patches of such sizes could clearly not be effectively inserted into skin using a single thumb. Since it is our opinion that patients will be more accepting of a large MN patch that can be applied by hand rather than an applicator device, we sought here, for the first time, to examine the ability of human volunteers to successfully use large MN patches.

## 2 Material and methods

### 2.1 Materials

Gantrez^®^ S-97 (Mw = 1.2 × 10^6^ Da), a copolymer obtained from the free acid of methyl vinyl ether and maleic anhydride polymers, was provided by Ashland (Tadworth, Surrey, UK). Poly(ethylene glycol) (PEG, molecular weight 10,000 Da) was purchased from Sigma-Aldrich (Steinheim, Germany). Parafilm M^®^, a flexible thermoplastic sheet (127 μm thickness) made of olefin-type material and used as a skin simulant for insertion studies, was obtained from BRAND GMBH (Wertheim, Germany). In order to build the MN patch, an adhesive dressing, Tegaderm™ (3M, St Paul, USA), and a pressure-responsive film, Pressurex-micro Green^®^ (Sensor Products Inc., Madison, USA) were used.

### 2.2 Fabrication of hydrogel-forming MN arrays

MN arrays were prepared using aqueous blends containing 15% w/w Gantrez^®^ S-97 and 7.5% w/w PEG 10,000. The formulation (0.5 g/cm^2^) was carefully syringed onto silicone micromoulds (14 × 14 needles on a 0.5 cm^2^ area, 600 μm needle height). The needle cavities were filled after applying positive pressure (3–4 bar) to the formulation for 15 min. MN arrays were left to dry for 48 h at room temperature and were then crosslinked by esterification through heating at 80 °C for 24 h (Donnelly et al., 2012a, Donnelly et al., 2014b, Larrañeta et al., 2015, Lutton et al., 2015b). The MN arrays were stored under ambient until assembled into large patches.

### 2.3 MN array characterization

MN were inspected using a Leica EZ4 D digital microscope (Leica, Wetzlar, Germany) and a Keyence VHX-700F digital microscope (Keyence, Osaka, Japan).

Attenuated total reflectance (ATR) Fourier transform infrared (FTIR) spectroscopy was used to evaluate the crosslinking of MN arrays. The IR spectra were recorded at room temperature using a FTIR Accutrac FT/IR-4100 Series (Jasco, Essex, UK) equipped with MIRacle™ software from 4000 to 600 cm^−1^, with a resolution of 4.0 cm^−1^. The obtained spectra were the result of averaging 64 scans.

Axial compression forces (*i.e.* forces applied perpendicular to the needle base) were applied to MN arrays in order to evaluate needle strength. A known load was applied to the MN arrays in axial compression mode of a TA.XT-Plus Texture Analyser (Stable Micro Systems, Surrey, UK). MN arrays were attached to the moving test probe of the Texture Analyser using double-sided adhesive tape. The MN arrays were then pressed against a flat block of aluminium at a rate 0.5 mm s^−1^ until a maximum force of 30 N per array was applied. The change in needle height was evaluated using the Leica EZ4 D digital microscope.

Parafilm^®^ M (PF) film was used as a skin simulant for MN insertion studies, as described previously (Larrañeta et al., 2014). MN arrays were inserted into a sheet assembled from 8 layers of Parafilm^®^ using the Texture Analyser, with the probe lowered onto the artificial membrane at a speed of 0.5 mm s^−1^, with an exerted force of 30 N per array, held for 30 s. The MN arrays were removed from the polymeric sheet after insertion, the PF sheet unfolded and the number of holes in each layer was evaluated using the Leica EZ4 D digital microscope. In order to ease the detection of the created holes in the PF layers, the sample was placed between two polarizer filters. The thickness of each PF layer was determined previously to be 126 ± 7 μm (Larrañeta et al., 2014) and this knowledge was used to calculate the percentage of MN successfully inserted as a function of depth.

### 2.4 Assembly of large patches from individual microneedle arrays

**Fig. 1:**
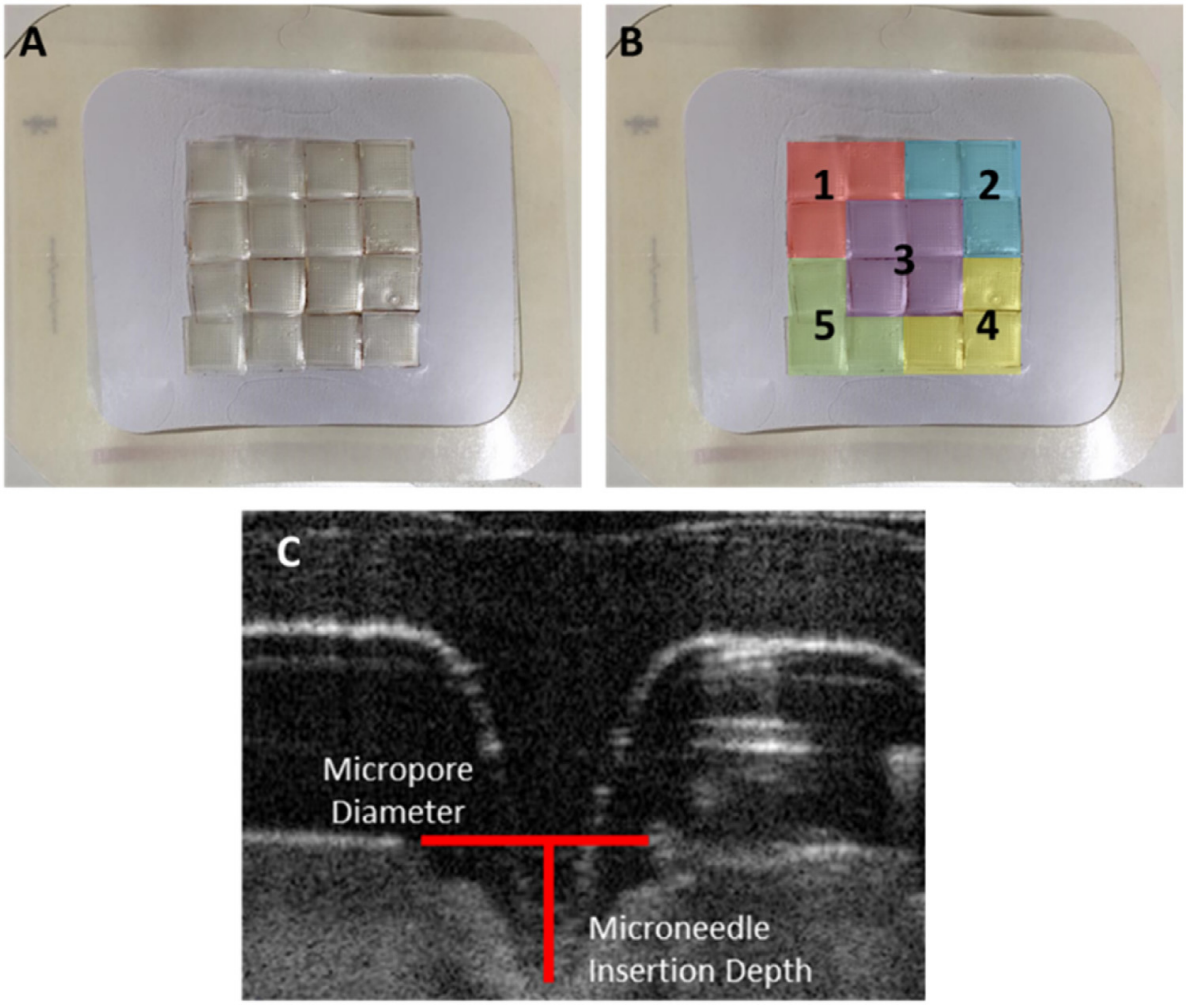
Tegaderm™ patch comprised of 16 individual MN arrays (A). MN patch numbered at the positions where the Optical Coherence Tomography (OCT) scans were taken (B). OCT image of a single MN penetrating a volunteer’s skin (C).

Large MN patches were assembled by using the adhesive properties of Tegaderm™ films (10 cm × 12 cm) to attach 16 individual MN arrays (Fig. 1). To the reverse, non-adhesive side of the assembled patches, the pressure-responsive Pressurex-micro Green^®^ indicator sensor films were attached using double-sided adhesive tapes. Pressurex-micro Green^®^ changes colour from white to red when pressures >20 N cm^−2^ are applied. Notably, colour changes are only seen on areas of the film where the pressure exceeds the cut-off. Below this, the microparticles from the red donor layer are not transferred to the white receiver layer. We have previously shown that pressures of approximately 10 N cm^−2^ are sufficient to successfully insert hydrogel-forming MN into human skin *in vivo*. Accordingly, Pressurex-micro Green^®^ serves as a useful patient feedback tool, confirming MN insertion. For larger MN patches, where correct insertion of each individual array will be required for consistent dosing, addition of such site-specific feedback will be vital.

### 2.5 Volunteer recruitment

Ten healthy volunteers (6 men and 4 women), aged between 20 and 30 years old, with no pre-existing skin conditions were recruited to the study by means of an email circular. Volunteers were asked not to apply cosmetic formulations to their upper arm or forearm 24 h prior to the study and to avoid hot showers/baths or exercise immediately before the study. Volunteers were provided with a patient information leaflet and a volunteer information sheet detailing the aims and objectives of the study, their contribution and, in particular, the risks associated and the confidentiality of the results obtained upon recruitment. The School of Pharmacy Research Ethics Committee, Queen’s University Belfast, approved this study. All volunteers were asked to confirm their fully-informed consent by signing an appropriate form prior to participating in the study (See Supporting Information). All data was anonymised and stored on firewalled servers in password-protected files and was scheduled for destruction 2 years after completion of the study. Only the researchers directly involved in the study had access to the data.

### 2.6 Patient information leaflet and counselling

The patient information leaflet (PIL) used in this study (See Supporting Information) was prepared with particular emphasis on existing PILs for transdermal patches’ (*e.g.* BuTrans^®^). Reference was made to PILs for other products (*e.g.* metred-dose inhalers) that require a more descriptive protocol and the inclusion of Figures to ensure volunteers would feel confident enough in applying the patches correctly.

The views of in-house microneedle researchers and patient-facing scientists involved in pharmacy practice research were considered when designing the PIL, to ensure appropriate format and content. The PIL was also then checked for best practice against the criteria laid out in “Quality Criteria for PILs” published by the UK’s Medicines & Healthcare Products Regulatory Agency (MHRA, 2016) to ensure it was of an appropriate standard. PILs are normally supplied with medicinal products and devices when the patient is expected to use them by themselves. Medicinal packs are required to include a PIL if the label does not contain all the necessary information. Upon dispensing medicinal products, such as our MN patches to a patient, it is normal practice for the pharmacist to provide counselling on how the product is to be used successfully.

A strategy for counselling every volunteer was developed, following discussion. This consisted primarily of details concerning MN fabrication and technology, how to hold and apply the patches (force required was described as “pressing an elevator button firmly”), what the pressure-indicating sensor film was used for, what exactly was going to be recorded using an optical coherence tomography instrument (VivoSight™ Topical Multi-Beam OCT Handheld Probe, Michelson Diagnostics Ltd, Kent, UK) and, finally, how to remove and dispose of the patch (Table 1).

### 2.7 Volunteer application protocol

The study was conducted at a controlled room temperature of 20 °C and a relative humidity of 45 ± 5%. The volunteers were acclimatised to the room for 10 min before starting the experiment. During their wait, subjects were presented with the PIL and volunteer information sheet and allowed to read about the application technique of the patches alongside counselling from the researcher(s).

Each application used a new MN patch. The four corners of where the patch was to be applied were marked on the volunteer’s upper arm. Before the volunteers applied the patches, baseline transepidermal water loss (TEWL) values were recorded at the edge of where the patch was to be applied and at the centre by carefully resting the TEWL probe (VapoMeter^®^, Delfin Technologies Ltd, Kuopio, Finland) on the application site, with the probe head vertical and perpendicular to the skin, to give an indication of skin barrier function. TEWL measures the outward diffusion of water through the skin and, as such, provides information on the barrier status (Vicente-Pérez et al., 2016). Measurement of TEWL is often used as an alternative to invasive measures of skin barrier integrity obtained by local excision or biopsy. Indeed, the technique has been frequently used in our previous MN-based studies, due to its patient-friendly non-invasive nature and capability for rapid and accurate data generation. TEWL measurements were taken, with values recorded for a period of 10–15 s and expressed in g m^−2^ h^−1^.

Each volunteer was then presented with the large MN patch within its package (moisture-impermeable, heat-sealable non-woven poly(ester) film, Transparent Film Products Ltd, Newtownards, UK) and asked to apply it to their upper arm, peel back the protective film on the receiving layer of the pressure-responsive film and apply pressure for 45s, following the steps detailed in the PIL. The pressure-responsive film was then peeled back, to ensure that the red colour was evenly distributed, hence indicating that sufficient and even pressure was applied by the volunteer to the patch.

The patch was numbered from one to five, at each corner and the middle, in order to be able to take five different scans of the MN penetrating the skin (Fig. 1B), using optical coherence tomography (OCT) (Fig. 1C). Once the subject had removed the patch from their arm, the TEWL was again measured at the edge and centre of where the patch had been applied.

The transepidermal water loss of the volunteer’s forearm was then measured and an individual MN array was applied by the volunteers to their forearm. Using their thumb, pressure was applied to the MN for 45 s. The pressure-responsive film was removed and again OCT was used to take a scan of the MN penetrating the volunteer’s skin. One more TEWL measurement was made to determine the difference in outward diffusion of water after subsequent removal of the MN.

Volunteers were revisited after 15 min, 1 h and 24 h to check for any adverse skin sensations or reactions and a digital picture (Nikon Coolpix™, Nikon, Tokyo, Japan) of their upper arm was taken at each time interval under controlled and consistent lighting conditions.

### 2.8 MN insertion parameters

Since TEWL does not give an indication of MN penetration depth, or the diameter of the created micropores, OCT technology was used to scan images of the MN penetrating the volunteers’ skin. OCT was used, as described previously (Donnelly et al., 2012a, Donnelly et al., 2014a), to study microneedle penetration depth and micropore diameter and to determine whether all 16 arrays of a large patch had successfully penetrated the *stratum corneum*.

Data was presented as means (±S.D.) of 5 replicate measurements of MN penetration depth and the diameter of the corresponding micropore for each of 5 microneedle arrays per patch. The MN penetration depth/micropore diameters were selected at random from the 196 penetrating microneedles comprising each array (each of the five scans taken). Quantification was performed using the imaging software ImageJ^®^ (National Institute of Health, Maryland, USA). The scale of the image files obtained was 1.0 pixel = 4.2 μm, thus allowing accurate measurements of the depth of MN penetration and the diameter of micropores created.

OCT was also used to scan images of the MN penetrating the skin of the volunteers’ forearms, after having applied the single MN array. Data was presented as means (±S.D.) of 10 replicate measurements of MN penetration depth and the diameter of the corresponding micropores of the single MN array. The MN penetration depth/micropore diameters were selected at random from the 196 penetrating MN in the array. Again, quantification was performed using ImageJ^®^.

### 2.9 Volunteer application force measurement

After having applied both the large MN patch and the individual MN array, the volunteers’ application/insertion force was approximated over a period of 45 s, using the Texture Analyser in tension mode, as described previously (Larrañeta et al., 2014). Three different readings were taken of the forces applied by each volunteer and the minimum, maximum and mean force (Larrañeta et al., 2014).

### 2.10 Questionnaire

A structured questionnaire, consisting of 7 fixed questions was developed. Questionnaires were designed after consulting with MN researchers and registered, patient-facing, pharmacists. With ethical committee approval and fully-informed consent, the paper-based written questionnaires were provided to the volunteers once the insertion/application study was complete. The questionnaire was used to evaluate the volunteers’ thoughts and feedback on the self-application of larger MN patches compared to individual MN and also their overall opinion on the potential of this technology and design (scale strongly positive to strongly negative 1–10). Quantitative data obtained was analysed using Microsoft Excel^®^ (Microsoft Corporation, Redmond, USA).

### 2.11 Statistical analysis

Statistical analysis was performed using the Wilcoxon Signed Rank test and Kruskal-Wallis test, as appropriate. In all cases, *p <* 0.05 denoted significance.

## 3 Results

### 3.1 Microneedle characterisation

**Fig. 2:**
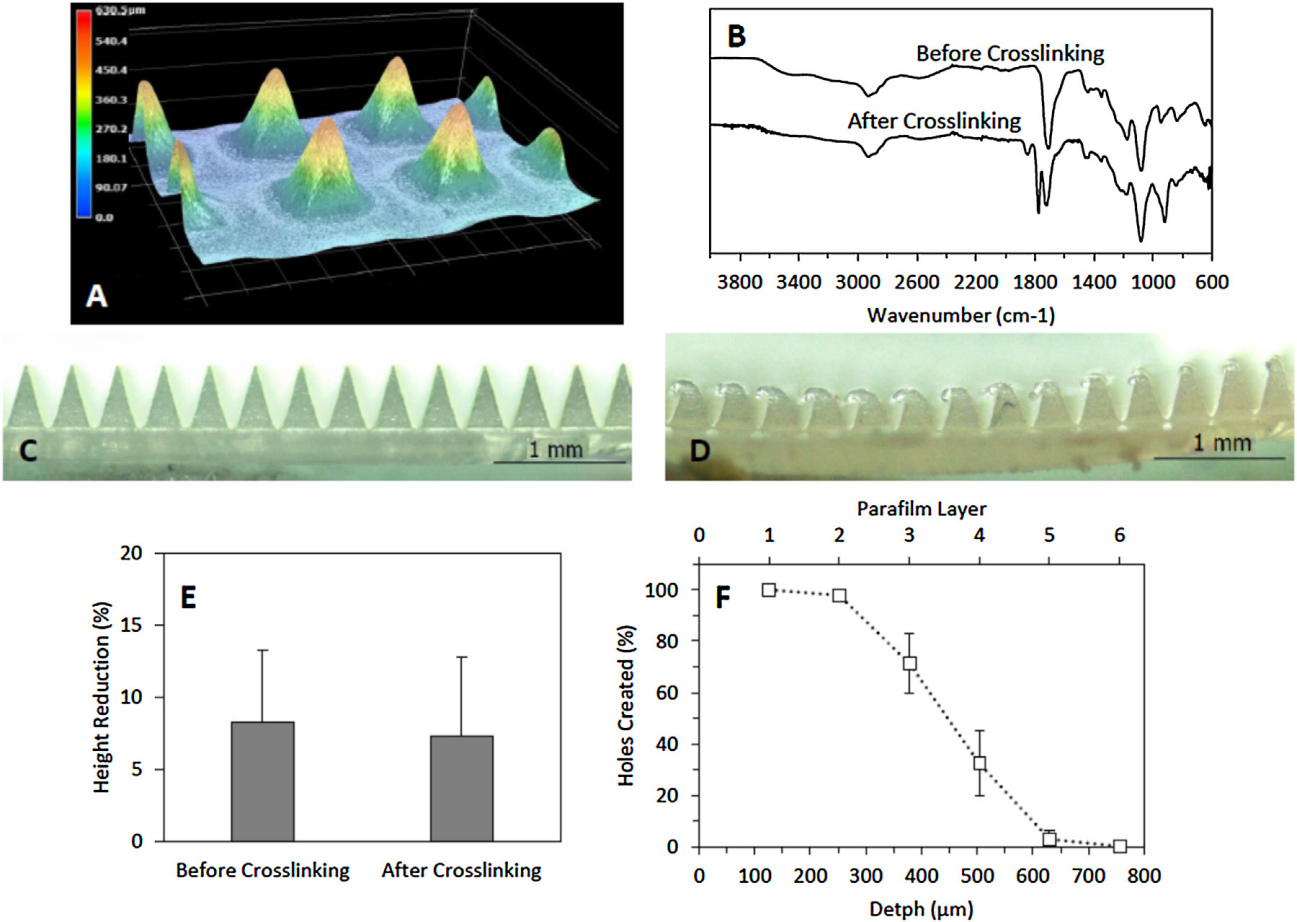
3D reconstruction of needle tips from a MN array (A). FTIR spectra of MN arrays before and after crosslinking (B). Microscopy images of MN arrays before (C) and after (D) compression study. Needle height reduction after compression (E). Insertion profile in Parafilm^®^ for MN (F). (Means ± SD, n = 3).

Before starting insertion studies, the manufactured MN arrays were tested. Optical microscopy was used to ascertain the geometry and morphology of the arrays. Fig. 2A shows a 3D reconstruction of some needle tips from one of the arrays, showing the pyramidal geometry.

A crucial step in manufacture of hydrogel-forming MN is the chemical crosslinking of the polymers that form the arrays. Infrared spectroscopy is the most convenient way to evaluate the crosslinking reaction. Fig. 2B shows the IR spectra of MN arrays before and after the crosslinking reaction. It is noticeable that the crosslinked arrays present a noticeable new carbonyl peak at around 1800 cm^−1^. This shows that the esterification reaction between Gantrez^®^ S97 and PEG took place.

MN should have sufficient mechanical strength to be inserted successfully in the skin without failure during insertion. The compression testing (Fig. 2C–E) shows that there is a needle height reduction after compressing them against an aluminium block (Fig. 2C and D). This reduction ranges between a 5 and a 15% of the original height (Fig. 2E). It should be noted, though, that the needles were compressed against a metal block and this situation is far from simulating the insertion of MN into the skin. This test was simply used to compare different batches of MN arrays and confirm consistency of manufacture, which it did. Finally, the insertion of the arrays was evaluated by using a validated skin simulant model (Larrañeta et al., 2014). The insertion profile (Fig. 2F) shows that the needles can theoretically reach insertion depths of 500 μm.

### 3.2 Microneedle self-application

**Fig. 3:**
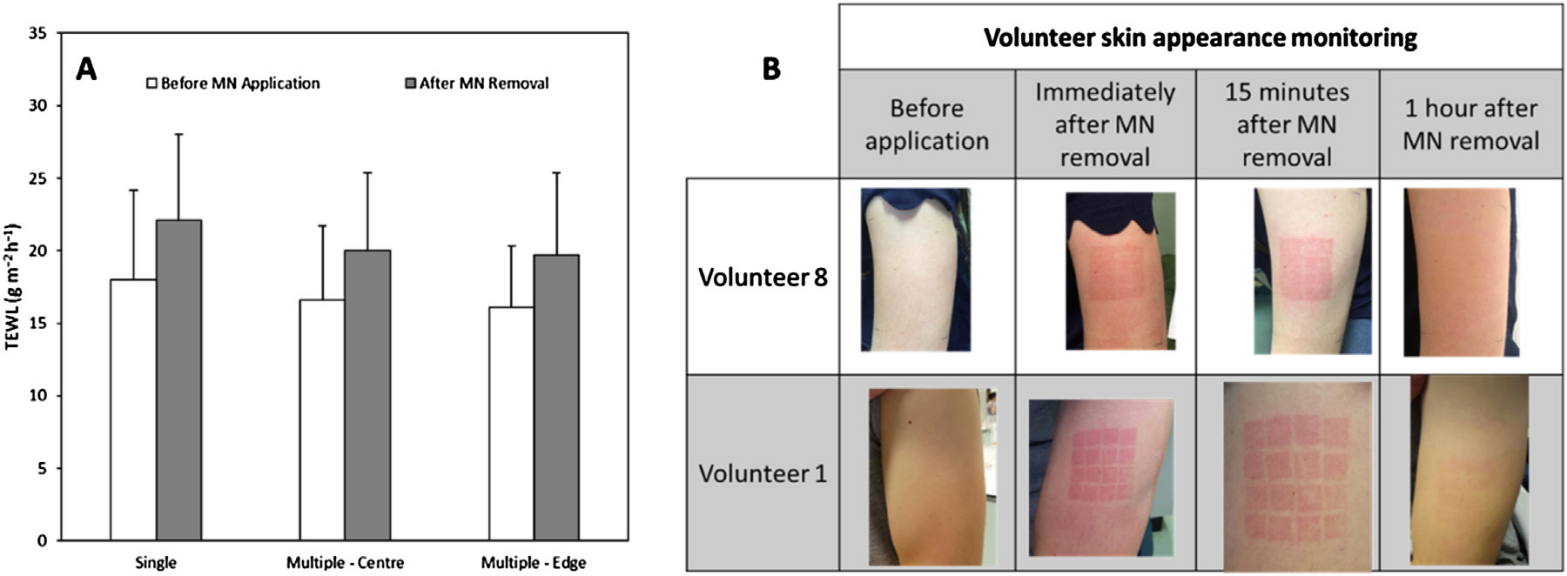
TEWL values before and after self-application of single MN arrays and patches comprised of multiple arrays (A). After the application of the multi-array patches TEWL was measured in the centre and at one edge of the region to which the larger patch was applied. Skin appearance following removal of larger patches in two representative volunteers (B).

Fig. 3A shows the post-removal increase in the TEWL values after volunteer self-application of a single MN patch and a patch comprised of multiple MN arrays. When patches comprised of multiple arrays were used, TEWL was measured in the centre of the application site and at one edge. The results showed that there is no significant difference between the application of single MN arrays and multi-array patches in terms of skin barrier disruption (*p* = 0.405). For large patches, the data shows that there is no influence from the measuring site (*p* = 0.083), in that the TEWL values obtained after MN removal at the centre of the application site are equivalent to those obtained from one edge of the application site.

The skin appearance of two representative volunteers can be seen in Fig. 3B. In both examples, it is noticeable that erythema can be found immediately after MN removal. After 15 min, the erythema begins to dissipate. After 1 h, erythema was not noticeable for the volunteers.

**Fig. 4:**
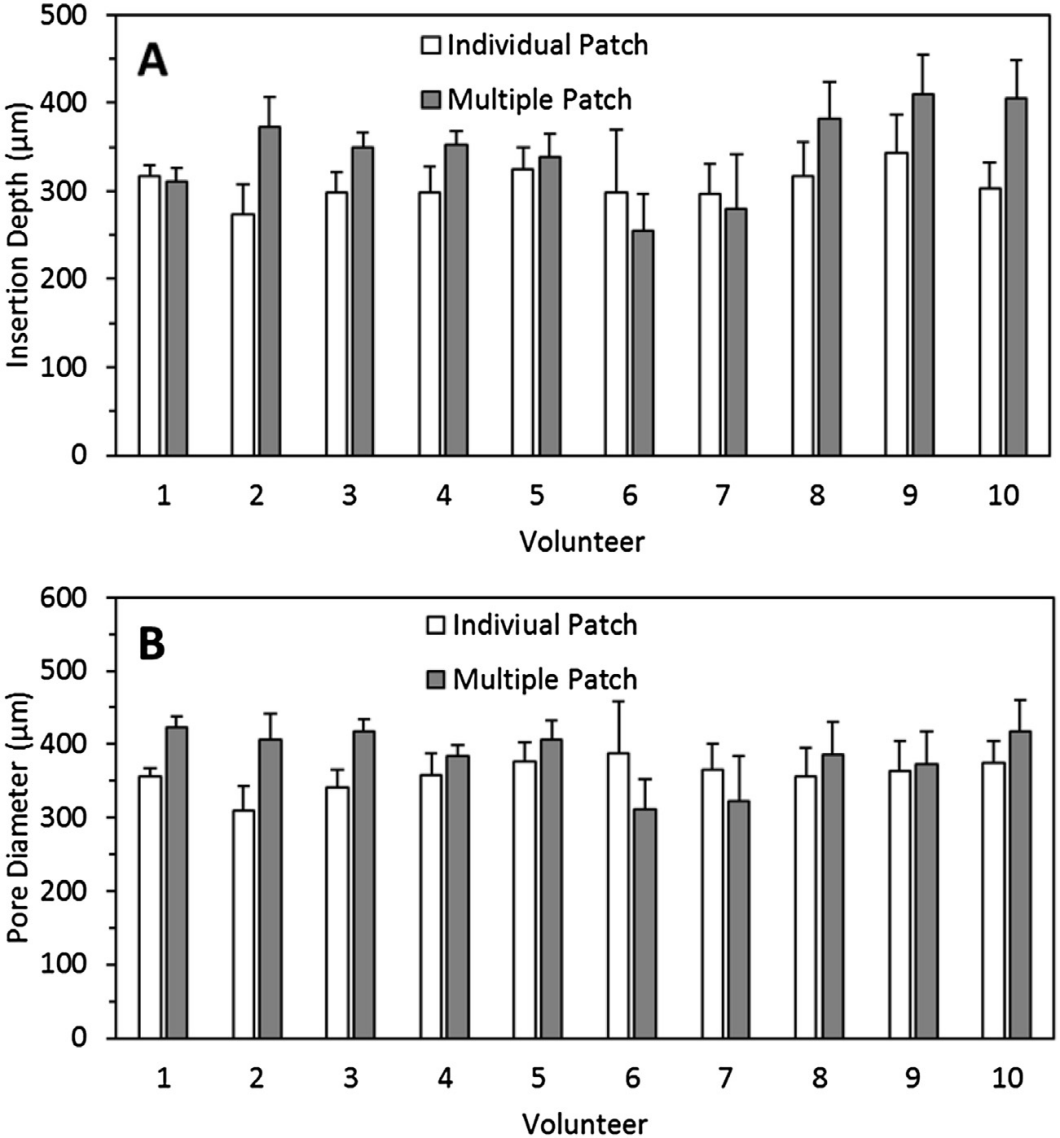
Insertion depth (A) and diameter of the created micropores (B) after the self-application of single and multi-array patches by 10 volunteers.

In order to ascertain the insertion of the arrays into the patient’s skin, OCT was used. Fig. 4A shows the mean insertion depths of a single MN array and a multi-array patch in the volunteer’s skin. The mean insertion depths ranged between 300 and 450 μm. As can be observed in Fig. 4A, there are no notable differences (*p *> 0.05 in each case) in insertion depths for MN of the larger patch and individual patch inserted by the same volunteer. Fig. 4B shows the diameter of the created micropores after MN insertion in the volunteers’ skin. The pore diameter ranged from 300 to 450 μm. Again, no statistically-significant differences were observed for any of the volunteers (p > 0.05 in each case) between the single and larger patches.

**Fig. 5:**
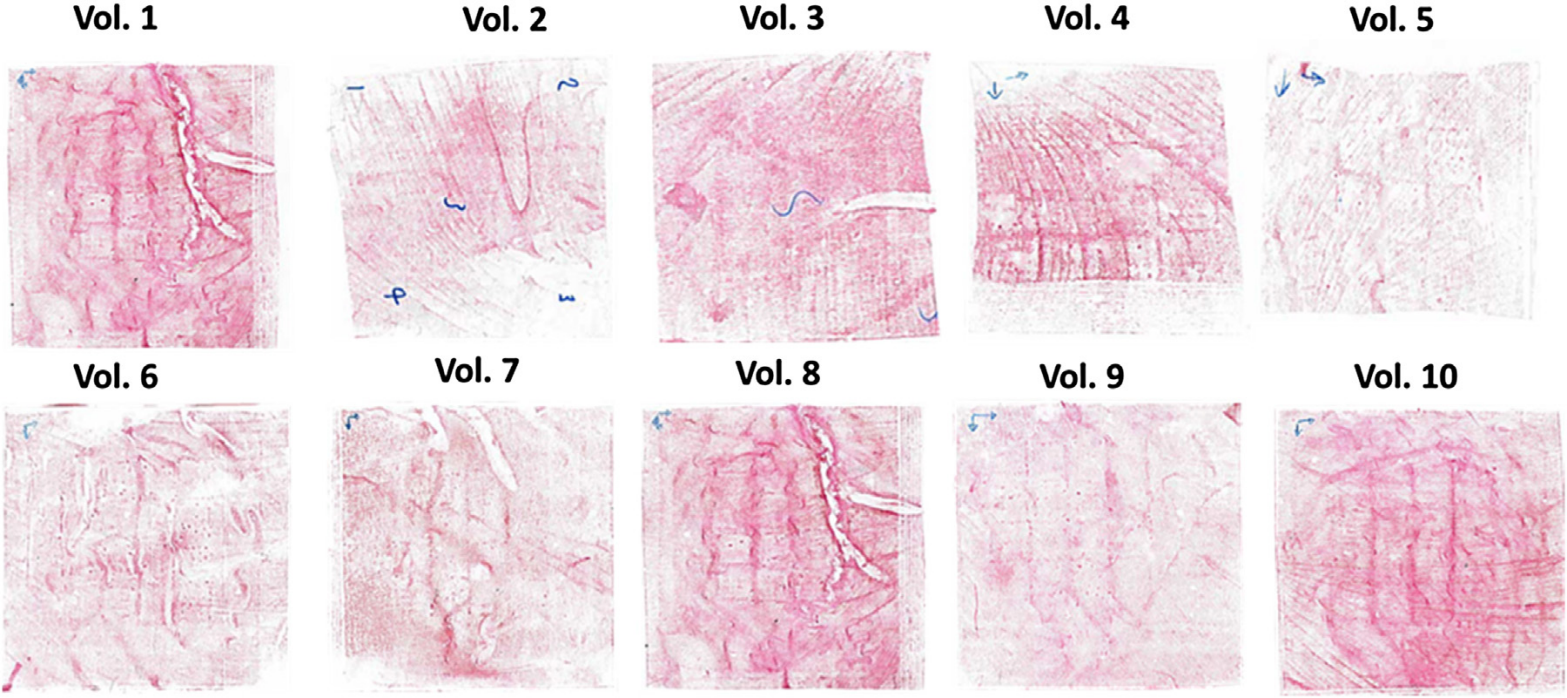
Scanned images of the pressure-indicating sensor films used as a low-cost insertion feedback mechanism during self-application of large MN patches by human volunteers.

Fig. 5 shows the pressure responsive films after application of large MN patches. It can be seen that, in all cases, the films turned red, indicating that sufficient pressure was applied over the entire region of the patch to allow successful skin insertion of MN.

**Fig. 6:**
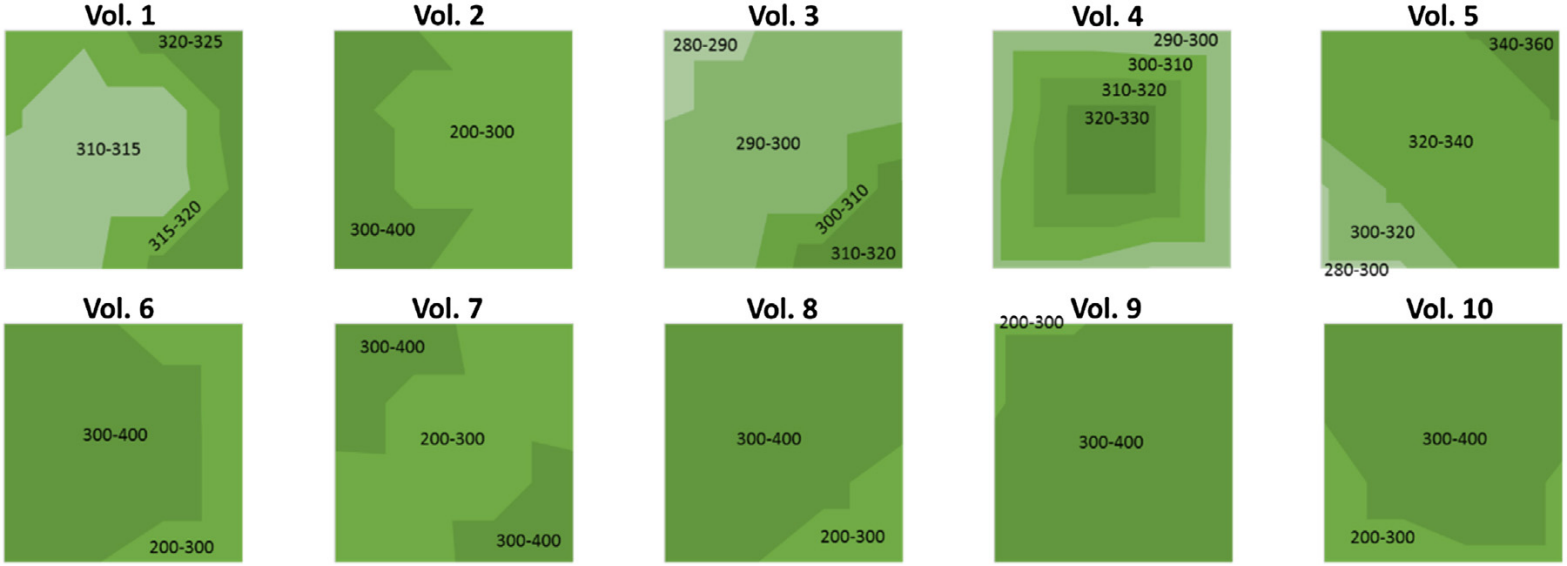
Insertion distribution of self-applied MN multi-array over the patch surface for all the different volunteers.

The insertion depth data presented in Fig. 4 represents mean insertion depths over the five specified regions of the multi-array patches. The insertion depth distribution over the entire patch surfaces can be seen in Fig. 6. It is interesting to note that insertion depths range between 200 and 400 μm over the surface of the patches. While insertion depth clearly varied between regions and between volunteers, it is important to realise that, in all measured cases, MN penetrated the *stratum corneum* and were inserted to a minimum depth of 200 μm.

**Fig. 7:**
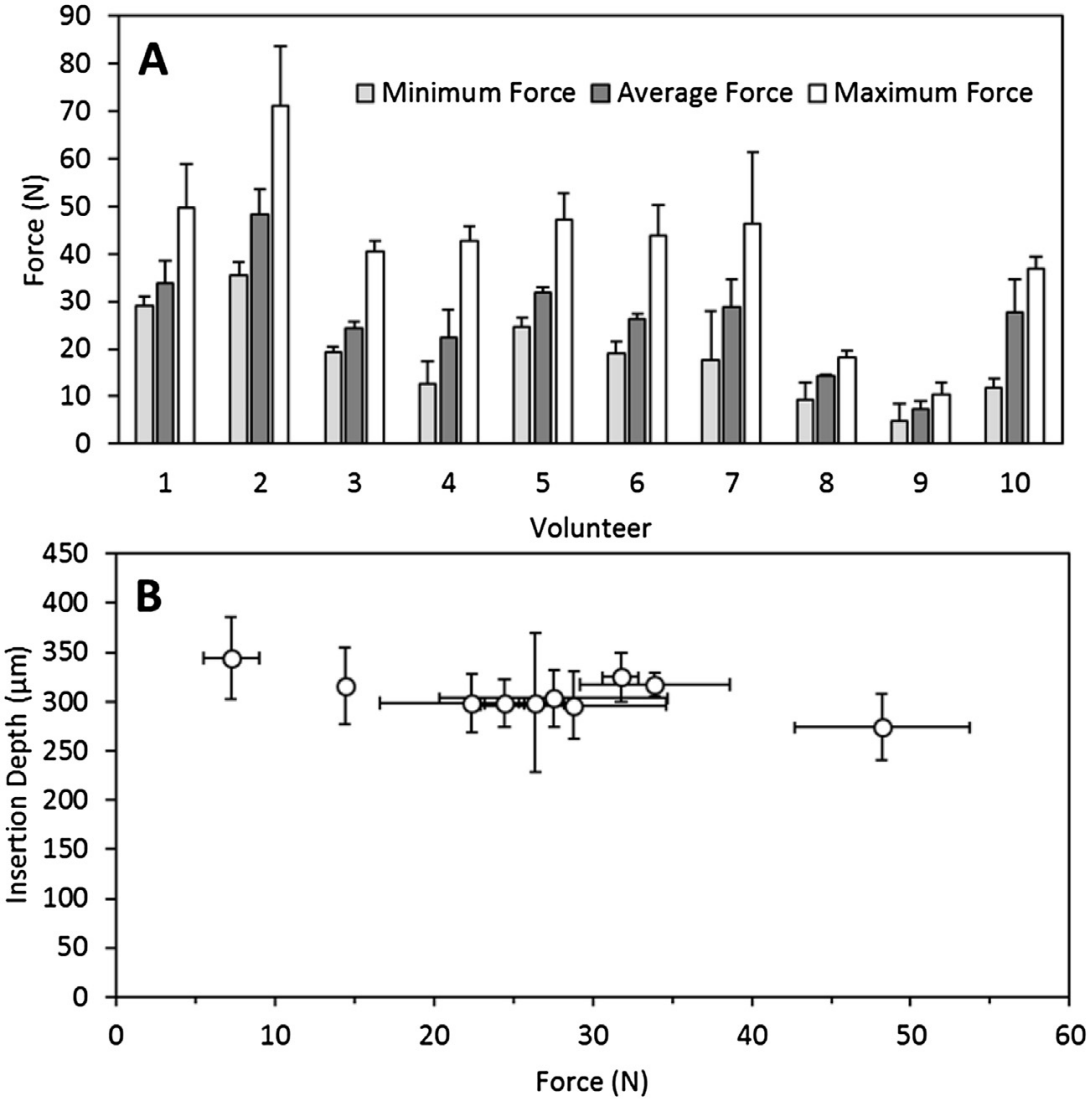
Maximum, minimum and mean forces applied by the volunteers when following the same instructions as for MN application/insertion (A). MN insertion as a function of the measured mean force applied by the volunteers (B). It is important to note than the insertion force and the insertion depth measurements were obtained in separate experiments.

Fig. 7A shows the maximum, minimum and average force applied by the volunteers when following the same instructions as for MN application and measured using the Texture Analyser. The distribution of forces was wide and some volunteers, such as Volunteer 1 and Volunteer 2, applied noticeably higher forces that the others. The lowest forces were applied by Volunteer 8 and Volunteer 9. Nevertheless, if one compares the applied forces with the mean insertion depths (Fig. 7B), it can be seen that insertion depth is not necessarily dependent upon the forces applied by these volunteers. It may well be that the minimum insertion force required for these arrays is considerably lower than the forces applied by the volunteers.

As can be seen from Table 2, the volunteers were generally positive about MN technology and most found application/insertion of both the larger MN patches and the individual MN array to be pain-free. The volunteers did find the individual array easier to use and some expressed concerns about inter-patient variability in application. However, 8 out of the 10 volunteers were confident that they had applied the large MN patches correctly themselves.

## 4 Discussion

Despite criticism about the speed of development and clinical translation, for a relatively new field, MN technology has actually made considerable progress since the first research work was published in 1998 (Henry et al., 1998). During this time, a vast number of types of MN arrays, including solid metal and silicon, dissolving polymeric, hollow and hydrogel-forming MN have all been investigated as delivery systems (Larrañeta et al., 2016c, Prausnitz, 2004, Tuan-Mahmood et al., 2013).

The focus of the field to date has largely been on vaccine delivery, meaning only small MN patches would be required for successful use in humans. While the earliest work was rightly centred on the fundamental science of delivery and understanding of the resulting physiological effects, recent years have seen enhanced emphasis on translation to patient benefit and commercial return, with several notable companies making progress towards commercialisation. Indeed, a number of studies examining patient usability (Birchall et al., 2011, Donnelly et al., 2014a, Vicente-Pérez et al., 2016), scaled-up manufacture and quality control testing (Larrañeta et al., 2015, Larrañeta et al., 2016b, Lutton et al., 2015b), sterile production (McCrudden et al., 2015a) and regulatory considerations (Lutton et al., 2015a) have now been published. MN with applications in improving skin appearance are now available commercially, though it is notable that these are not approved drug products (McCrudden et al., 2015b).

Our own translationally-focussed work has suggested that dissolving MN arrays and hydrogel-forming MN arrays can both potentially be used for the delivery of high drug doses sufficient to elicit therapeutic effects in humans, providing patch size is appropriate (Donnelly et al., 2014b, McCrudden et al., 2014). As described previously, hydrogel-forming MN arrays seem to be the most suitable candidate for the delivery of higher doses of therapeutic molecules. They are not limited by the amount of drug loaded inside the needle tips, as the drug is incorporated in an attached patch-type reservoir (Donnelly et al., 2012a). By optimising the formulation, this type of system is capable of delivering high doses of low potency drugs without depositing measurable amounts of polymer in skin (Donnelly et al., 2014b). MN patch size has never before been a consideration, due to the potency of most vaccines. However, in order to deliver therapeutically-relevant doses of most drugs, much larger MN patches will be required, even for hydrogel-forming MN. Ideally, such patches would not exceed the sizes of already marketed transdermal patches, so as to be acceptable to patients. They should also be able to be reliably and reproducibly applied by all patients, following suitable instruction or counselling. Successful translation of such a technology would clearly create a significant opportunity for industry in terms of expanding the size of the transdermal market, which has now been in stagnation for several years, with few new drug candidates in the pipeline (Watkinson et al., 2016). Accordingly, our focus in the present study was to ascertain, for the first time, how successfully larger MN patches could be applied by human volunteers.

Here, we have shown that multi-array large MN patches can be successfully prepared by assembling together single arrays using an adhesive patch. The needles themselves exhibited good mechanical properties, as they did not break during direct compression and showed deeper insertion profiles than those reported by us previously (Lutton et al., 2015b), possibly due to the move from conical to pyramidal needle designs.

The use of larger MN patches inevitably changes the way in MN are applied/inserted. To date, MN have been inserted into skin manually, or by using an applicator device (Singh et al., 2011, Vicente-Pérez et al., 2016). Such approaches work relatively well for small patches, as the applied force is easily focused over a small area. For a larger patch, several fingers, or the palm of the hand will be required to ensure even insertion of the needles distributed over the entire area of the patch. A rigid applicator device would most likely not be suitable for patches greater in area than around 5 cm^2^, due to the curvature of the human body. This also means that the baseplate upon which the MN of a single-unit large MN patch are formed, or the adhesive backing to which numerous individual smaller MN arrays are attached to make up the larger MN patch, must exhibit a degree of flexibility to avoid cracking. For manual application of larger patches, a means of patient feedback will be essential. It is conceivable that a patient will not apply sufficient force to the entire area of a large patch and so inconsistent needle penetration will result, leading to inconsistent drug dosing.

Here, we showed that the large MN patches, comprised of 16 individual arrays assembled on a flexible adhesive backing were self-inserted by human volunteers as effectively as single MN arrays. The pressure-responsive indicator film changed colour in each case, providing instant feedback on successful insertion. Despite variation in applied pressures and differences in depths of insertion at different sites across the patch areas, the *stratum corneum* barrier was always overcome, with minimum insertion depths of 200 μm observed in all cases. Volunteer feedback was positive, providing encouragement for further development of this approach.

## 5 Conclusion

This work has shown that large MN patches can be produced in a simple manner and effectively self-inserted by human volunteers provided with appropriate instruction and that a low-cost pressure-responsive film can provide accurate real-time confirmation of successful insertion. Our work continues to centre on exemplification of delivery of therapeutically-relevant doses of drug substances using our hydrogel-forming MN technology, with scaled-up manufacture to GMP standards and clinical translation a strong current focus.

## Figures and Tables

**Table 1:** Researcher counselling points on microneedle patch application protocol to human volunteers.

Point 1	“Using an alcohol wipe, rub your upper arm gently, to ensure the area of application is clean to provide clear images using optical coherence tomography.”
Point 2	“Feel that the microneedle patch is at the bottom of the packaging. The pressure-responsive film should be facing upwards. Gently pull open the Tegaderm™ patch ensuring you do not press on the microneedle arrays when doing so, as this may damage the microneedles”
Point 3	“Place the microneedle patch with the microneedles facing into your upper arm and ensure the Tegaderm™ adhesive border adheres to your skin. Gently pull the protective layer off the receiving layer of your pressure-responsive film and fold over the red layer. Press on the area of your patch firmly and evenly. Hold this pressure for around 45 s”
Point 4	“Stop applying pressure and peel off the red film of your pressure-responsive film to ensure you have applied even pressure across the whole patch. The researcher will then sequentially take scans of five different areas of the patch.”
Point 5	“Gently peel off the Tegaderm™ film, pulling the adhesive film away from you to minimise removal pain. Place the used patch in the original packing it came in and dispose of”
Point 6	“Any skin redness is only temporary and is not a cause for concern”

**Table 2:** Views and opinions obtained from 10 human volunteers using a structured questionnaire.

Questions asked about large microneedle patch application	Number of volunteers that stated answer
Q1. Do you believe this method of microneedle application has potential for drug delivery?	
YES	10

Q2. Did you find the PIL helpful and easy to understand?	
YES	9
NO	1

Q3. What limitations or problems do you think may be encountered with the general patient population using large microneedle patches?	
1. Inter-patient variability in applying pressure or skin thickness	10
2. Not confident the drug had entered the body uniformly	3
3. Potential for misuse and abuse	0
4. Possible high cost of microneedles compared to hypodermic injection	3
5. Pain associated with administration could result in low patient compliance	0

Q4. What advantages do you think larger microneedle patches have compared to individual arrays?	
1. Less painful than hypodermic injection	9
2. Possibility of self-administration	10
3. Less bleeding	8
4. Less tissue damage	7
5. Less needle stick injuries	9
6. Reduced fear of injection	10
7. Reduced frequency of administration compared to individual arrays	7

Q5. Please rate how painful the application of (1) the individual microneedle array and (2) the larger microneedle patch was using the provided visual analogue scale, where 0 = no pain and 10 = very painful	
0–3	9
4–7	1
8–10	0
0–3	9
4–7	1
8–10	0

Q6. Please rate how easy the application of (1) the individual microneedle array and (2) the larger microneedle patch was using the provided visual analogue scale, where 0 = very easy and 10 = very difficult	
0–3	9
4–7	1
8–10	0
0–3	7
4–7	2
8–10	1

Q7. Indicate on the provided visual analogue scale how confident you are that you applied the large microneedle patches correctly, where 0 = not confident at all and 10 = very confident	
0–3	0
4–7	2
8–10	8
