# Supplementary material for: Successful application of large microneedle patches by human volunteers
Source: Int J Pharm. 2017 Apr 15;521(1-2):92–101. doi: 10.1016/j.ijpharm.2017.02.011 (PMC5364775; doi:10.1016/j.ijpharm.2017.02.011)
Supplement: Supplementary file 1 [file mmc1.docx]

**VOLUNTEER CONSENT FORM**

**Title of Project:** Exploring the self-application of a large patch of multiple microneedle arrays in vivo compared to a single microneedle array

**Name of Researchers: Anastasia Ripolin, James Quinn**, School of Pharmacy, Queen’s University, Belfast

**Please initial box**

1. I confirm that I have read and understand the information sheet for the above study being undertaken. I have had the opportunity to consider the information, ask questions and have had these answered satisfactorily.
2. I understand that my participation is voluntary and that I am free to withdraw at any time without giving any reason.
3. I understand that the data collected during the study will be assessed by

Professor Ryan F. Donnelly, James Quinn and Anastasia Ripolin.

1. I agree that the data obtained during the study can be retained anonymously up to

2 years in password protected electronic format and accessible to James Quinn, Anastasia Ripolin, Eneko Larraneta and Professor Ryan F Donnelly. I also agree that it can be published in international journals or in the project of James Quinn and Anastasia Ripolin.

1. I agree to take part in the above study.
2. *Following this study:*

Would you be willing to return and complete further study in August? YES/NO

Date : ______________

Name of volunteer (PRINT NAME) : __________________________________

Signature : _____________________________________________________
