# Supplementary material for: Successful application of large microneedle patches by human volunteers
Source: Int J Pharm. 2017 Apr 15;521(1-2):92–101. doi: 10.1016/j.ijpharm.2017.02.011 (PMC5364775; doi:10.1016/j.ijpharm.2017.02.011)
Supplement: Supplementary file 2 [file mmc2.docx]

**Volunteer Information Sheet**

**Study Title:** Exploring the self-application of a large patch of multiple microneedle arrays in vivo compared to a single microneedle array

**Invitation to Participate**

You are invited to take part in a research study. Before you decide, it is important to understand why the research is being done and what it will involve. Please take time to read the following information carefully. Feel free to talk to others about it.

**What is this study about?**

This study is based on the application of polymeric microneedle (MN) patches to the skin. MNs are tiny projections that painlessly by-pass the outer layer of the skin, creating microscopic pathways for the transport of drugs to the skin’s blood supply. Following removal of the MN patch, these micro-pores close very quickly allowing skin barrier function to return to normal. This innovative strategy has been used to enhance the delivery of drugs which do not have the necessary properties to cross the skin. For example, MN patches, through extensive investigation have facilitated the delivery of molecules such as vaccines or insulin.

The following study will be focusing on the application of large patches consisting of multiple microneedle arrays and will require volunteers to compare the ease and effectiveness of these patches compared to a single MN array. Optical coherence tomography (OCT) is a non-invasive imaging technique used to look inside the skin and determine the extent of penetration by the MNs. It is CE marked and approved for use in humans.

**What is the purpose of this study?**

MNs offer a safer and less painful method of transdermal delivery compared to a hypodermic needle. It therefore may be used in the future by members of the public. In light of this, we intend to assess the ability of volunteers to successfully and correctly apply both a single array and a bigger patch of microneedles to their skin. The overall perception of larger microneedle patch application will therefore be established thus furthering this research area in the School of Pharmacy at Queen’s University, Belfast.

The MN patch applied to the volunteers will be prepared from FDA-approved polymeric materials, namely, 20% Gantrez® S 97 and 7.5% PEG 10,000 [Poly(ethylene glycol)]. These polymers are widely used in a diverse range of pharmaceutical and cosmetic formulations, for topical, oral and parenteral use and are known to be non-toxic and non-irritant.

**Why have I been invited?**

We need 10 volunteers in this study. Through convenience sampling, our aim is to successfully obtain sufficient scientific data on the insertion of MN patches as well as volunteer application feedback on the overall perception of the larger patches.

**Do I have to take part?**

No. We will provide all relevant information and then let you deicide. You will then be asked to sign a consent form to indicate agreement of participation. However, you are free to withdraw at any time, without giving a reason.

**What will happen to me if I take part?**

By consenting to take part in this study, you agree to apply a single microneedle array plus a larger patch consisting of 16 individual arrays to your forearm and allow a member of the research team to measure the skin penetration after MN insertion, using OCT and skin irritation and skin barrier disruption using TEWL (transepidermal water loss). The researcher will tell you which patch to apply first. Application time of each MN is 30 seconds. Following this, you will then complete a short questionnaire.

**What are the possible disadvantages and risks of taking part?**

There are no disadvantages, side effects or risks to you taking part in this study. Your time is all that is required to participate. Previous studies have shown that you may feel a slight sensation of pressure when applying individual MN arrays, however larger patches have not yet been investigated.

**What are the possible benefits of taking part?**

There are no clinical benefits for you participating in this study. However, your participation will benefit future research in the School of Pharmacy at Queen’s University, Belfast.

**Will my participation in this study be kept confidential?**

Yes. We will follow ethical and legal practice and all information about you will be handled in confidence. The details of data collection, storage and use are detailed later.

**What will happen if I decide at a later date not to participate in this study?**

You are free to withdraw at any time of this study. If you withdraw, we will not use any data collected up to your withdrawal. It will immediately be destroyed.

**What do I do if I have a problem?**

If you have a concern about your participation in this study, contact Professor Ryan F. Donnelly, Chair in Pharmaceutical Technology (Tel: 028 9097 2251).

**How will my participation in this study be kept confidential?**

All information collected during the course of the research will be kept strictly confidential. Each volunteer will be randomly assigned a volunteer number (01, 02, etc) and this, along with the volunteers gender, will be the only information about them retained. Data obtained, in terms of results from clinical photographs will be associated with each volunteer number. The anonymised data will be processed using Excel and will be password protected for a period of 2 years, to allow completion of this current research project. Volunteer details (except volunteer numbers and genders) will not appear in the project or in any publications arising from the work. Any paper work relating to this study will be kept in a locked filing cabinet. All data relating to this study, including any paper work will be destroyed either by deletion of electronic files or by incineration after a 2 year period. Results will be reviewed only by Professor Ryan F. Donnelly, Anastasia Ripolin, James Quinn and Eneko Larraneta as they work together on publications.

**Who is funding this study and what will happen to the results of this research?**

The project is funded by School of Pharmacy, Queen’s University Belfast. Results obtained will be written as a scientific paper and in a journal publication.

**Who has reviewed this study?**

The Research Ethics Committee of the School of Pharmacy at Queen’s University, Belfast has previously granted approval for such measurements to be obtained by this research group in this manner.

**Further information and contact details**

If you require further information or further advice about this research project, please contact either of the individuals listed below:

*Researchers :* James Quinn Tel: 07842662477 , Anastasia Ripolin 07849291853

*Project Supervisor :* Professor Ryan F. Donnelly Tel: 028 9097 2251

**Thank you for considering to participate in the research study and taking the time to read this information.**
