# Supplementary material for: Successful application of large microneedle patches by human volunteers
Source: Int J Pharm. 2017 Apr 15;521(1-2):92–101. doi: 10.1016/j.ijpharm.2017.02.011 (PMC5364775; doi:10.1016/j.ijpharm.2017.02.011)
Supplement: Supplementary file 3 [file mmc3.docx]

**Volunteer Questionnaire**

Q1. Do you believe this method of microneedle application has potential for drug delivery?

Yes No

Q2. Did you find the PIL helpful and easy to understand?

Yes No

Q3. What limitations or problems do you think may be encountered with the general patient population using large microneedle patches? (You may tick more than one box)

1. Inter-patient variability in applying pressure or skin thickness
2. Not confident the drug had entered the body uniformly
3. Potential for misuse and abuse
4. Possible high cost of microneedles compared to hypodermic injection
5. Pain associated with administration could result in low patient compliance

Q4. What advantages do you think larger microneedle patches have compared to individual arrays?

1. Less painful than hypodermic injection
2. Possibility of self-administration
3. Less bleeding
4. Less tissue damage
5. Less needle stick injuries
6. Reduced fear of injection
7. Reduced frequency of administration compared to individual arrays

Q5. Please rate how painful the application of (1) the individual microneedle array and (2) the larger microneedle patch was, on the following scales:


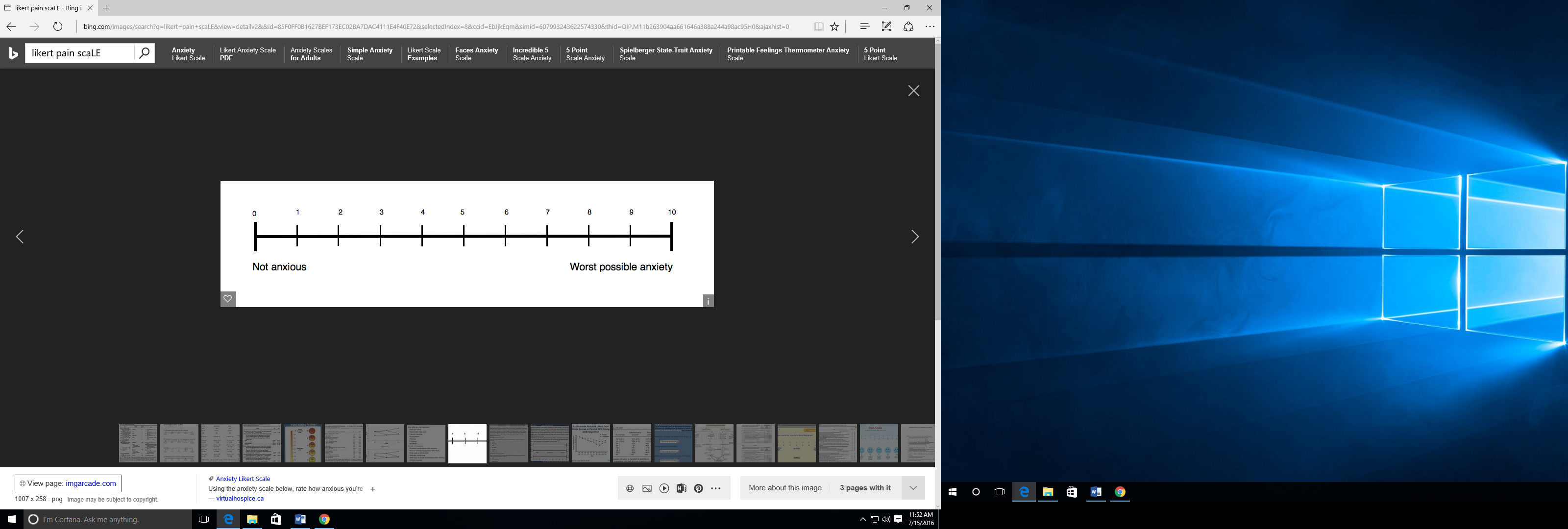


No pain Very painful


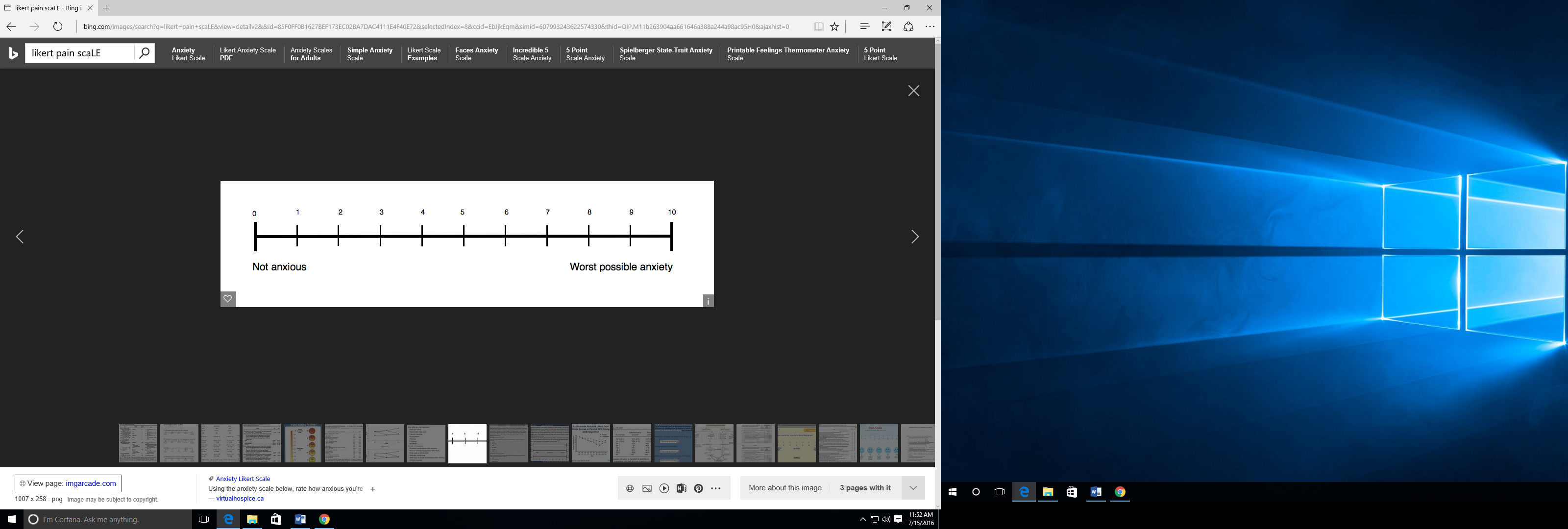


No pain Very painful

Q6. Please rate how easy the application of (1) the individual microneedle array and (2) the larger microneedle patch was, on the following scales:


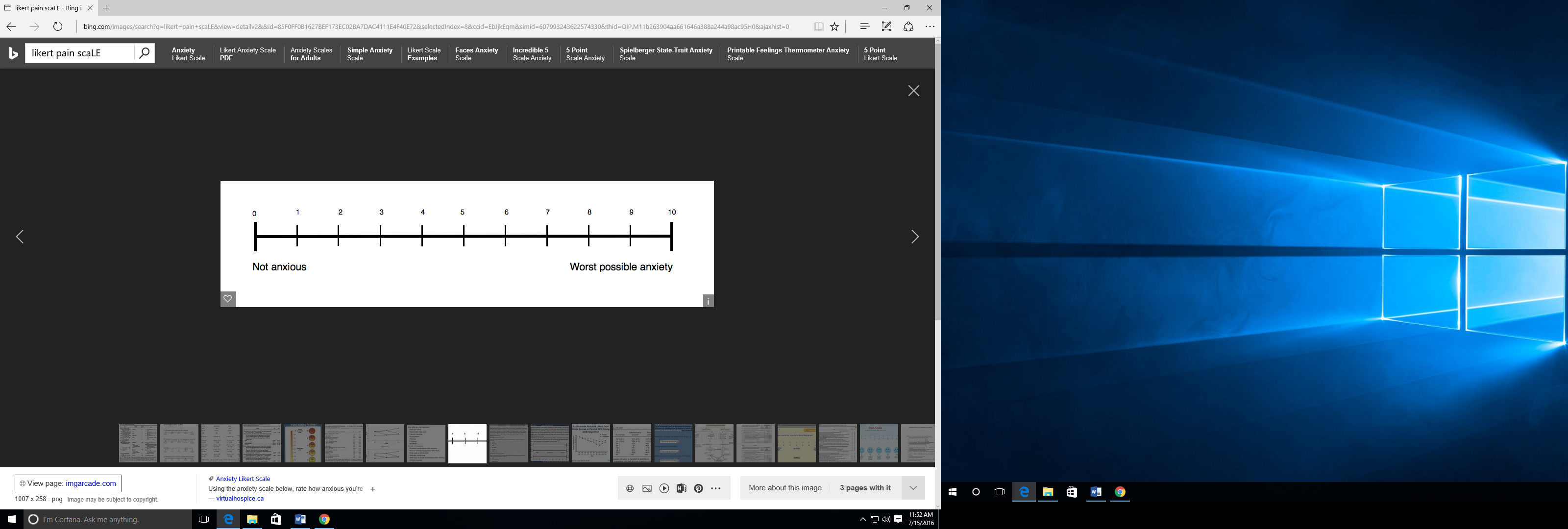


Very easy Very difficult


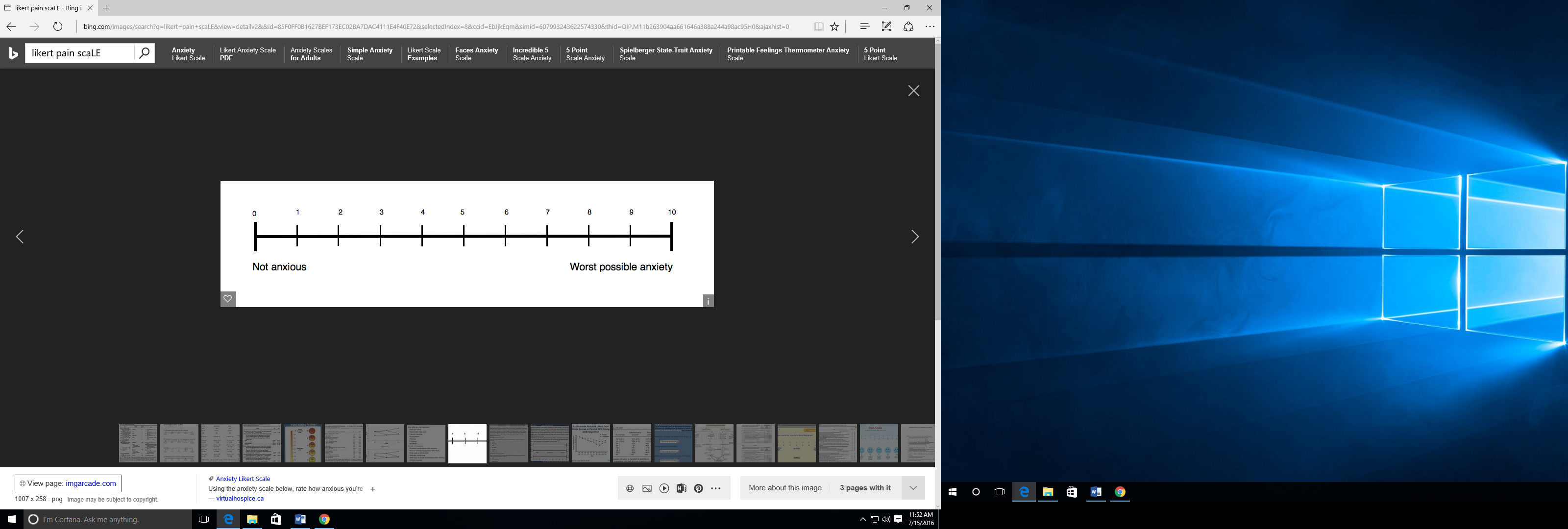


Very easy Very difficult

Q7. Are you confident you applied the large microneedle patches correctly?


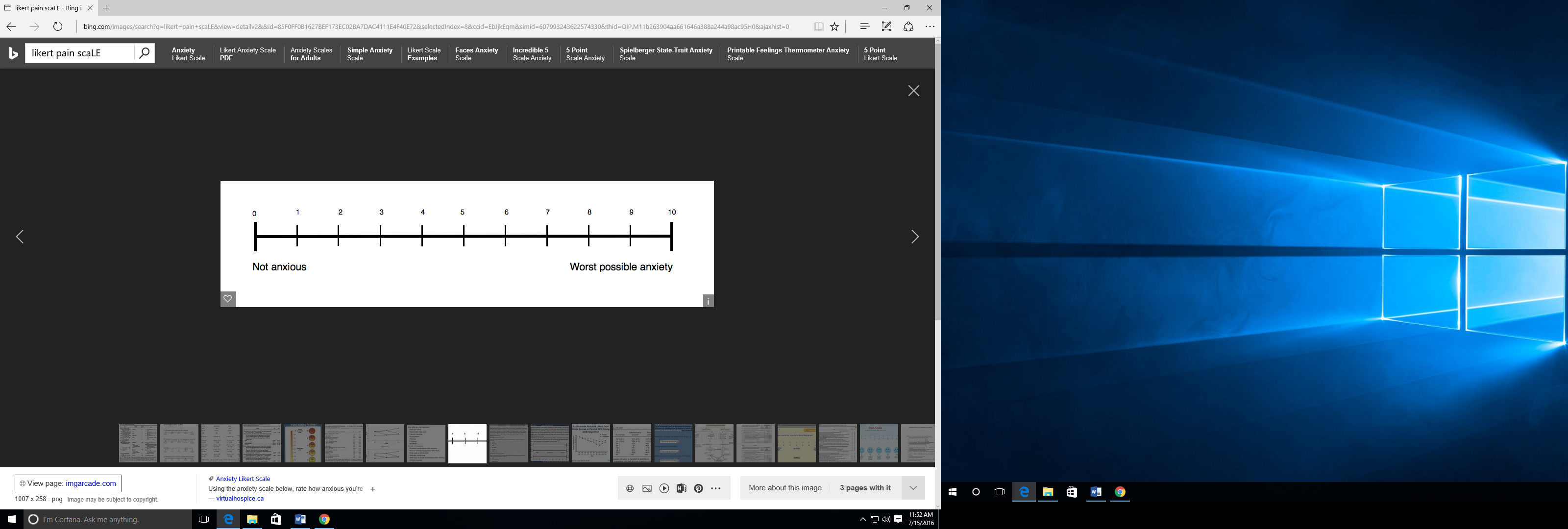


Not confident at all Very confident
