# Supplementary material for: Successful application of large microneedle patches by human volunteers
Source: Int J Pharm. 2017 Apr 15;521(1-2):92–101. doi: 10.1016/j.ijpharm.2017.02.011 (PMC5364775; doi:10.1016/j.ijpharm.2017.02.011)
Supplement: Supplementary file 4 [file mmc4.docx]

**Microneedle Patch**

*Information for the user*

**Please read this leaflet carefully before applying the patch:**

- Keep this leaflet. You may need to read it again.
- If you have any further questions, ask a member of the research team (details are provided at the end of the leaflet).
- This patch is for your use only, do not pass it on to others.

**In this leaflet:**

1. What *microneedle patches* are and what they are used for

2. Where to apply the microneedle patch

3. How to apply the microneedle patch

4. Possible side effects

5. How to store the microneedle patch

6. Further Information

**1. What *microneedle patches* are and what they are used for**

Microneedle patches are a new form of patch that can be used to deliver medicines across the skin and to the rest of your body.

A microneedle patch consists of tiny projections, approximately half a millimetre in height, arranged on a small base, which forms the patch for application to your skin (Fig. 1). When applied, the microneedles can cross the outer layer of your skin, creating pathways which allow medicines to be transported. Once removed, the tiny holes close rapidly and skin function returns to normal. Bigger microneedle patches (much like transdermal patches) consisting of multiple individual microneedle arrays are currently been investigated, with the potential of administering higher doses of medicines via the skin.

Fig. 1: Pictures showing the typical s­­­­ize of microneedle patches

Microneedle patches have a number of advantages:

- The application process is painless with no risk of bleeding;
- They are an alternative to a ‘traditional’ injection, eliminating needle phobia
- They provide an alternative to an oral tablet. This may be particularly useful for those who have to take numerous tablets or have swallowing difficulties, for example, older adults.
- Microneedle patches may not need to be applied as often as other drug preparations have to be used

**2. Where to apply the microneedle patch**

Apply the patch to your upper arm (see Fig. 2). The arm used will be opposite to your dominant hand, for example, the hand that you write with. Therefore, if you are right handed, you should apply the patch to your left arm.


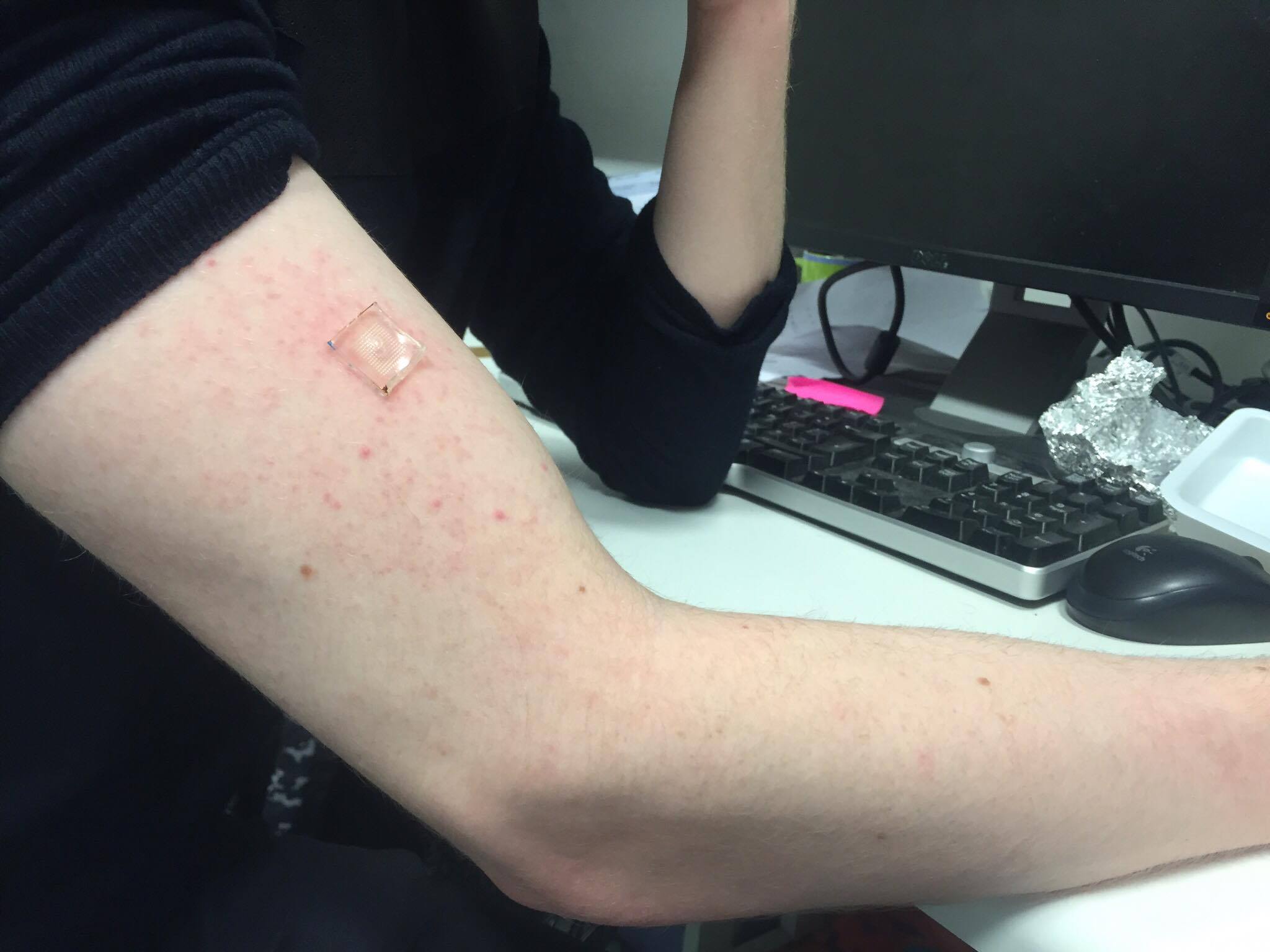


Fig. 2: Area of the body where microneedle patch is to be applied (upper arm)

**3. How to apply the microneedle patch**

Prepare the skin:

- Wash and dry your hands before application.
- Choose an area of non-irritated, intact skin on your upper arm. Avoid skin which is red, irritated or has any other blemishes, for example, large scars or moles.
- Ensure the area of skin where the microneedle patch is to be applied is hairless or nearly hairless. If no suitable hair free sites are available, the hairs should be cut off with scissors. Do not shave them off.
- Use the antiseptic wipe provided to clean the area of skin chosen.
- Wait until your skin is completely dry and cool before applying.

Open the pouch:

- Each patch is sealed in its own pouch.
- Feel that the microneedle arrays are at the bottom of the pouch before opening, as this will reduce the chance of breakage.
- Carefully tear or cut off the edge of the pouch completely (if you use scissors; cut where indicated on the packaging).
- Take the patch out and apply it immediately (following the instructions below).
- Retain the empty pouch in order to dispose of the patch later.

Apply the patch:

- You may find it easier to rest your arm on a flat surface when applying the patch.
- Ensure that the patch is held with the microneedles pointed downwards, against the skin. Try not to touch the microneedles before application.

There are two different patches; one contains an individual microneedle array which is 1.1cm in height and 1.1cm in width when they are trimmed. The other patch is one large microneedle patch, it contains the same type of array but there are 16 of the arrays arranged in a square shape. Please follow the appropriate instructions for the application of each patch as explained below. Both patches can be applied to the same upper arm. Only one microneedle patch will be applied at one time.


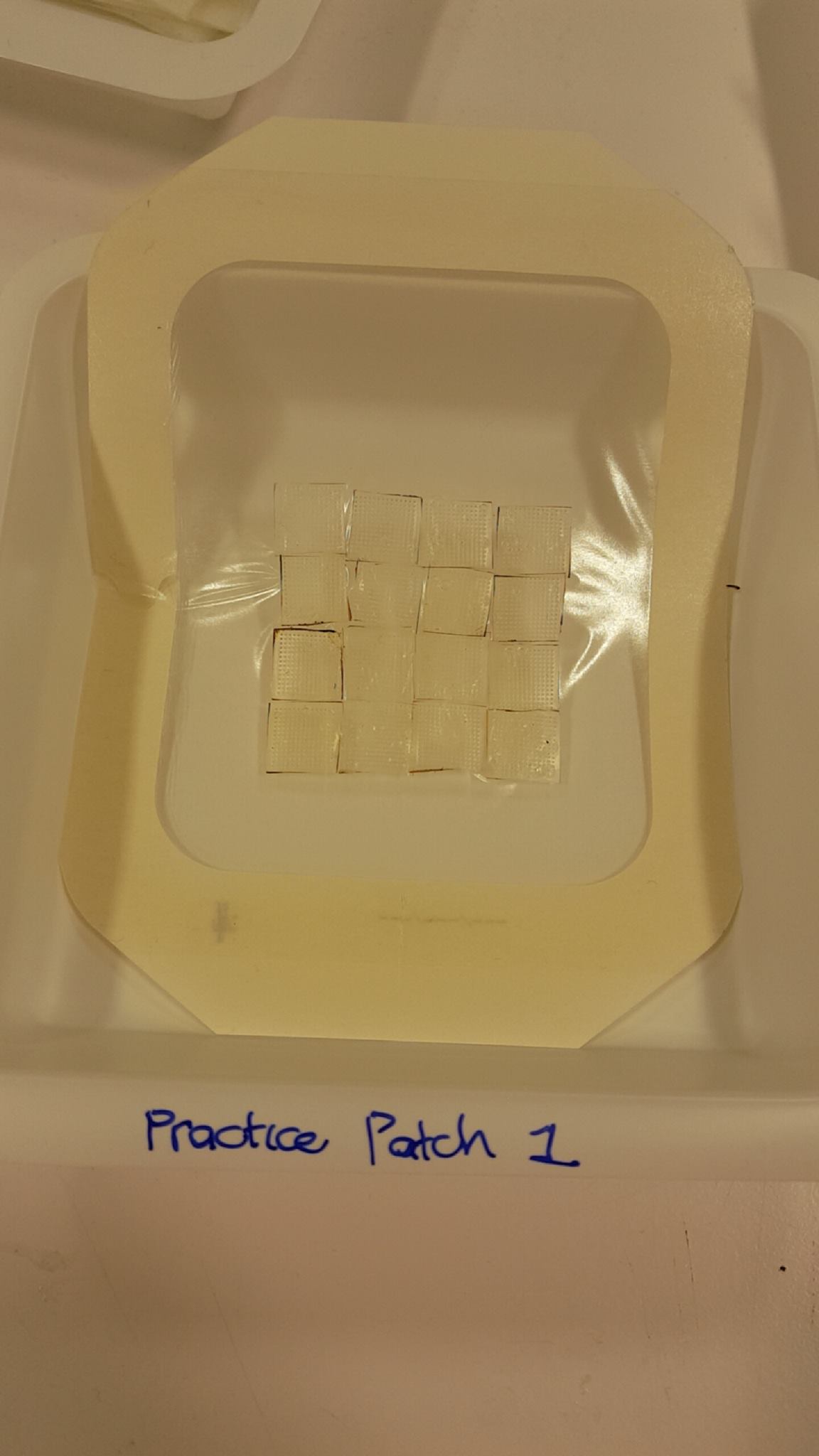

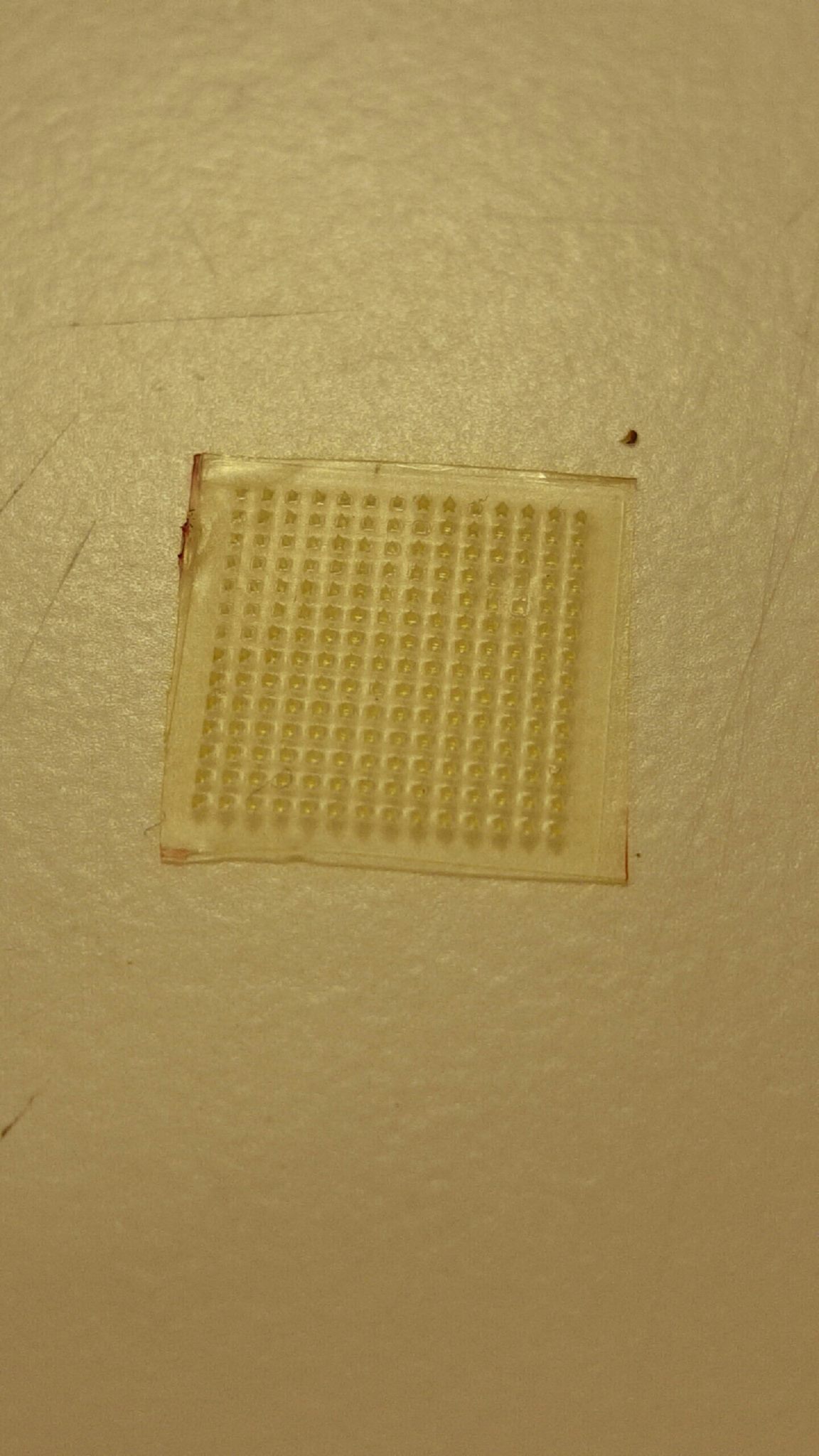

**Fig 3.** An individual microneedle array and a patch containing 16 microneedle arrays

**Patch** *with* **individual microneedle array:**

**STEP 1**

The sticky side of the patch is covered with a protective paper. Carefully peel off half the paper. Try not to touch the sticky part of the patch.

**STEP 2:**

Stick the patch on the area of skin of your upper arm you have chosen and remove the remaining paper.

**STEP 3:**

The pressure indicating sensor film consists of 2 layers (red and white) as shown below. Remove protective covering from the lower white layer. Allow the red layer to return to its position but DO NOT apply pressure. Once pressure is applied, the dye from the red layer will be transferred to the white layer, indicating that a sufficient pressure has been applied to successfully insert the microneedles.


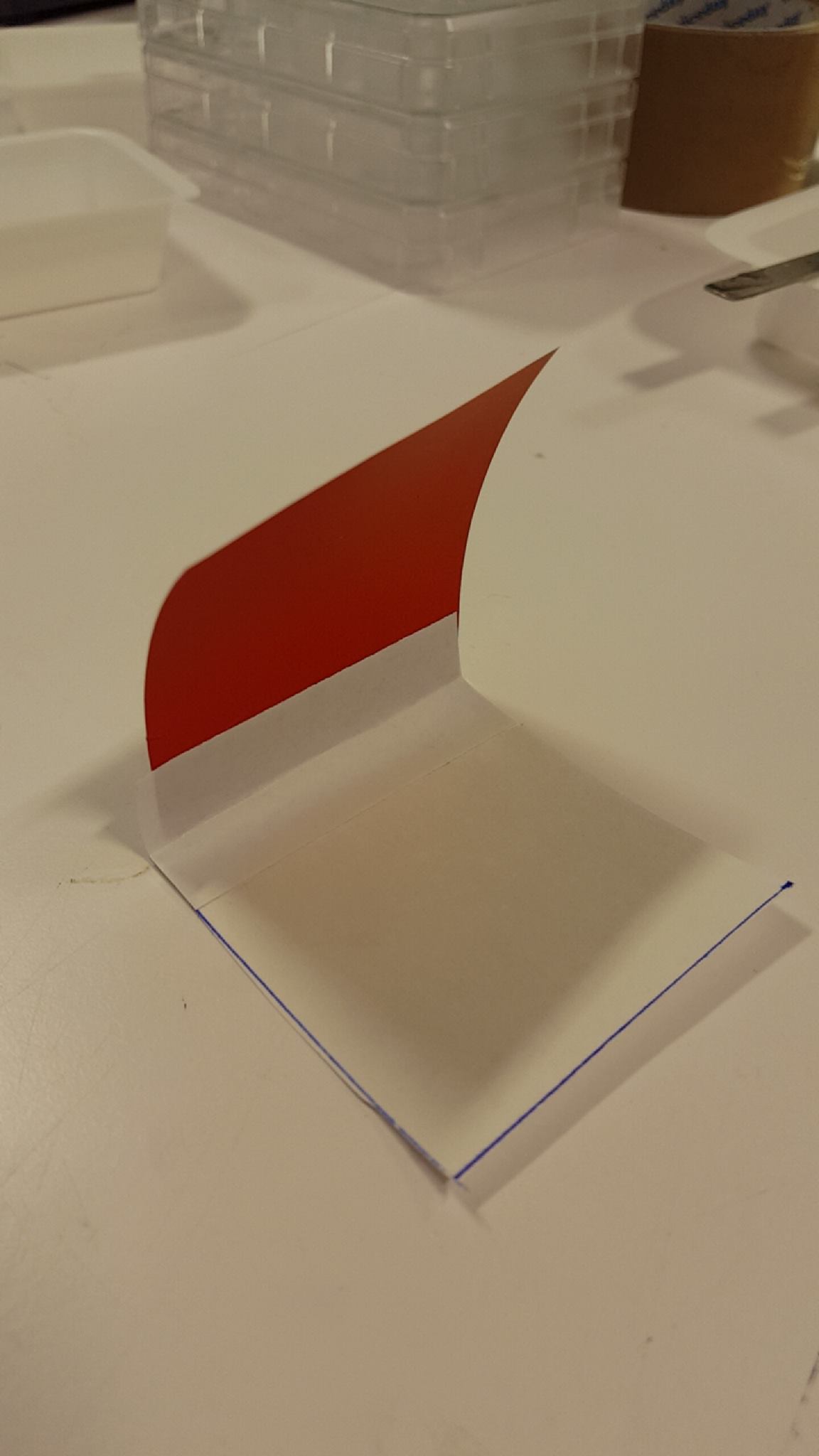


**Fig 4.** Pressure indicating sensor film

**STEP 4:**

Press the patch firmly against your skin with your thumb. Apply firm pressure on the back of the patch to ensure insertion of the patch into the skin.

- - Ensure the pressure is applied downwards, against the skin.
  - The amount of pressure required may be described as ‘pressing a button on an elevator’ or ‘putting a stamp on a postcard.’
  - Do not apply too much pressure as this may cause discomfort or possibly break the patch.
  - Hold the patch for 30 seconds (a timer will be available) and then release pressure.
  - Ensure patch remains in arm. In the unlikely event that your patch falls off, do not use the same patch again. Stick a new one on (see start of section 3 of the leaflet again).

**STEP 5:**

- The researcher will carefully remove the pressure indicating sensor film by cutting it from the rest of the patch. You will now see the microneedles. The researcher will then separate the lower layer from the top layer. There will be a colour change to red on the lower white layer, indicating appropriate pressure has been applied. The red layer removed can be disposed of in the general waste (see ‘Disposal’ section).

**Large patch** *with* **multiple microneedle arrays:**

- - Repeat Step 1 and Step 2 (see above).

**STEP 3:**

Press the patch firmly against your skin using the palm of your hand.

- - Apply firm pressure on the back of the patch to ensure even distribution of pressure and even insertion of the microneedles into the skin.
  - Ensure the pressure is applied downwards, against the skin.
  - Hold the patch for 30 seconds (a timer will be available) and then release pressure.
  - Ensure patch remains in arm. In the unlikely event that your patch falls off, do not use the same patch again. Select a new patch and start from Step 1 again.

**STEP 4:**

The researcher will carefully remove the pressure indicating sensor film by cutting it from the rest of the patch. You will now see the microneedles. The researcher will then separate the lower layer from the top layer. There will be a colour change to red on the lower white layer, indicating appropriate pressure has been applied. The red layer removed can be disposed of in the general waste (see ‘Disposal’ section). The researcher will ask your permission to hold your arm in order to orientate your arm such that the OCT laser can observe if the microneedles have been inserted into the arm.

Disposal

- - Remove the patch from the skin.
  - Fold the patch with the sticky side inwards.
  - Return the patch (and red layer as explained above) to the pouch and dispose of in the general waste.
  - Wash your hands, preferably with antibacterial soap after application.

**4. Possible side effects**

Side effects are rare with this patch as there is no active ingredient or medicine contained. The skin may appear red or itchy, but this should subside within a short time period, trials have shown that this is normally within one hour of insertion. If there is any prolonged redness, speak to a member of the research team (contact details are provided at the end of the leaflet).

**5. How to store the microneedle patch**

Keep out of reach and sight of children.

Store the microneedle patch in a cool dry place. Do not store the microneedle patch above 25°C.

**6. Further Information**

**What do *microneedle patches* contain?**

There is no active ingredient or medicine in the patches used for experimental purposes. The needles are each 600 microns in height and each patch contains 196 microneedles in total, in a 14 x 14 arrangement.

The patch is composed of;

- - 20% Gantrez^®^ S 97 [poly(methyl vinyl ether/maleic anhydride)]
  - 7.5% PEG [poly(ethylene glycol)] 10,000

These ingredients are known to be non-toxic and non-irritant; they are used in a number of common applications, including pharmaceutical and cosmetic formulations for consumption, use on the skin and injection.

Pressurex-micro^®^ Green pressure indicating sensor films are produced by Sensor Products Inc, Madison NJ, USA

**This leaflet was prepared in July 2016.**

If you require further information or further advice:

**Contact Information**

Project Supervisor: Professor Ryan F. Donnelly

Researchers: James Quinn, Anastasia Ripolin

**School of Pharmacy**, **Queen’s University, Belfast** 97 Lisburn Road, Belfast BT9 7BL

Telephone: +44 (0)28 9097 2086 (or +44 (0)28 9024 7794) Email: pharmacyinfo@qub.ac.uk
